# M7G-related molecular subtypes can predict the prognosis and correlate with immunotherapy and chemotherapy responses in bladder cancer patients

**DOI:** 10.1186/s40001-023-01012-x

**Published:** 2023-02-02

**Authors:** Deng-xiong Li, De-chao Feng, Xiao-ming Wang, Rui-cheng Wu, Wei-zhen Zhu, Kai Chen, Ping Han

**Affiliations:** Department of Urology, Institute of Urology, West China Hospital, Sichuan University, Sichuan Province, Guoxue Xiang #37, Chengdu, 610041 China

**Keywords:** m7G, Bladder cancer, Biomarker, Chemotherapy, Immunotherapy

## Abstract

**Background:**

N7-methylguanosine (m7G) is closely associated with tumor prognosis and immune response in many cancer types. The correlation between m7G and bladder cancer (BC) needs further study. We aimed to orchestrate molecular subtypes and identify key genes for BC from the perspective of m7G.

**Methods:**

RNA-seq and clinical data of BC patients were extracted from TCGA and GSE13507 datasets. The patients were subtyped by “ConsensusClusterPlus” and “limma.” The clusters were validated by the Kaplan‒Meier curves, univariable and multivariate Cox regression models, the concordance index, and calibration curves. The immunotherapy response was evaluated by immune checkpoints, immune infiltration, TIDE score, and IMvigor210 cohort. Genomics of Drug Sensitivity in Cancer was utilized to predict the chemotherapy response between the clusters.

**Results:**

The m7G-related cluster was ultimately established by EIF4G1, NUDT11, NUDT10, and CCNB1. The independent prognostic value of the m7G-related cluster was validated by the TCGA and GSE13507 datasets. The cluster was involved in immune-associated pathways, such as neutrophil degranulation, antigen processing cross-presentation, and signaling by interleukins pathways. Meanwhile, cluster 2 was positively correlated with many immune checkpoints, such as CD274, CTLA4, HAVCR2, LAG3, PDCD1, and PDCD1LG2. The cluster 2 was significantly correlated with a higher TIDE score than the cluster 1. Furthermore, in the IMvigor210 cohort, patients in the cluster 1 had a higher response rate than those in the cluster 2. Patients in the cluster 2 were sensitive to many chemotherapies.

**Conclusions:**

We successfully determined molecular subtypes and identified key genes for BC from the perspective of m7G, thereby providing a roadmap for the evolution of immunotherapy and precision medicine.

**Supplementary Information:**

The online version contains supplementary material available at 10.1186/s40001-023-01012-x.

## Introduction

Bladder cancer (BC) ranks as the 10th most commonly diagnosed carcinoma and caused 213,000 deaths globally in 2020 [1]. According to the American Cancer Society estimation, BC is predicted to take fourth place among newly diagnosed cancer cases among American men [2]. There are significant differences in morbidity between sexes. However, although the incidence of BC in females is low, the prognosis is worse than that in males [3]. Radical cystectomy (RC) is the standard treatment for limited BC. However, the health expenditure of BC and considerable morbidity caused by RC have brought heavy economic, living, and mental burdens to BC patients. To improve these situations, many therapies have been performed clinically, such as surgery, chemotherapy, immunotherapy, and radiotherapy [4, 5]. Despite various therapies, BC is still prone to recurrence, progression and even death, which may be due to its high genetic heterogeneity [6]. Under these circumstances, clinicians are striving to identify new powerful therapeutic targets. Novel immunotherapy is an outstanding representative of these efforts. Furthermore, to fully take advantage of the existing treatments, a powerful biomarker also requires much work from researchers, which can help determine the optimal treatment option [5]. With a useful prognostic biomarker, doctors can decide whether aggressive therapies, such as RC, should be performed.

N7-methylguanosine (m7G), one of the prevalent posttranscriptional modifications of RNA, is positively charged and produced by the addition of a methyl group at position N7 of riboguanosine [7]. In addition to participating in efficient pre-mRNA splicing and recruitment, the m7G cap is also required binding to the nuclear cap-binding complex [8]. Moreover, m7G can also occur on the caps of mRNAs and internally in mRNAs. Compared with unm7G-modified mRNAs, internal m7G-modified mRNAs have higher translation efficiency [9]. The core functional structure of m7G is the methyltransferase-like 1 (METTL1)/WD repeat domain (WDR4) complex, which can regulate RNA processing, metabolism, and function. Prior studies have noted the essential role of WDR4 in benign diseases, such as Galloway-Mowat syndrome [10], speech and language delay [11], and microcephalic primordial dwarfism [12]. Recently, m7G has been reported to participate in tumorigenesis and can predict the prognosis and treatment outcome of tumors [13]. Abnormal changes in m7G influence the occurrence and progression of malignant tumors. In glioblastoma, a study constructed a signature by four m7G-related genes (TMOD2, CACNG2, PLOD3, and TMSB10) and could predict the prognosis of glioblastoma patients [14]. Li et al. [15] identified m7G-related clusters that could predict the prognosis of hepatocellular carcinoma patients. Furthermore, in the field of urological tumors, three studies demonstrated the prognostic value of m7G-related mRNAs, lncRNAs and miRNAs in clear cell renal cell carcinoma [16–18]. Meanwhile, M7G-related genes could influence the immune infiltration of prostate cancer [19, 20]. However, a few studies have discussed the correlation between m7G and BC through single m7G-related gene and review [13, 21]. The role of m7G in BC needs to be further explored. Therefore, this evidence inspired us to identify the relationship between m7G and BC.

To explore and prove this correlation, we collected BC data from online databases to orchestrate molecular subtypes and identify key genes for BC from the perspective of m7G to provide a roadmap for the evolution of precision medicine. Then, various methods were employed to validate the clusters.

## Materials and methods

### Microarray data and molecular subtypes

We extracted the RNA-seq and clinical data of BC patients from the Cancer Genome Atlas (www.gdc.cancer.gov, TCGA) to analyze the expression and function of m7G-related genes. In terms of prognosis analysis, patients with postoperative survival times shorter than 30 days were excluded. For external validation, GSE13507, including clinical information of BC patients, was used to evaluate the prognostic value of the m7G-related clusters. The m7G-related genes were collected from a combination of the literature [19], which selected 42 m7G-related genes by screening previous reports and the GSEA database (http://www.gsea-msigdb.org/gsea/index.jsp).

All 42 m7G-related genes were incorporated into the lasso regression model, which could apply penalties to these genes. The penalty parameter (λ) for the model was determined by tenfold cross-validation following the minimum criteria. Four genes (EIF4G1, NUDT11, NUDT10, and CCNB1) were selected. Then, the patients in the TCGA and GSE13507 datasets were subtyped by the R packages “ConsensusClusterPlus” and “limma” using the four genes. The consensus matrix k value denoted the number of m7G-related clusters. Then, based on a linear regression-based algorithm and a label propagation algorithm, GeneMANIA (www.genemania.org) [22] was employed to predict the interacting proteins of EIF4G1, NUDT11, NUDT10, and CCNB1.

### Validation of the m7G-related cluster

Depending on the survival results of the TCGA and GSE13507 datasets, the prognostic value of the m7G-related cluster, including overall survival (OS), cancer-specific survival (CSS), and progression-free survival (PFS), was evaluated by Kaplan‒Meier curves. Due to lacking the data of PFS time and CSS time data, we did not plot these Kaplan‒Meier curves in GSE13507. Furthermore, the OS in different clinical subgroups, such as male, T2_4 stage, and age > 70 years subgroups, was also compared by the Kaplan‒Meier curves. The independent prognostic value of the cluster was estimated by univariable and multivariable Cox regression models based on the TCGA and GSE13507 datasets. In detail, according to the results of the univariable Cox regression model, a multivariable Cox regression model was constructed using factors with *P* < 0.1.

According to the results of above multivariable Cox regression models, two nomograms were carefully established based on the cluster and clinical parameters in the TCGA and GSE13507 datasets. For validation, the concordance index (C-index) and calibration curves were used to validate the reliability and accuracy of the nomograms.

### Functional enrichment analysis

First, based on TCGA data, Gene Ontology (GO) enrichment analysis was used to explore the possible function of the cluster in molecular function (MF), biological process (BP), and cellular component (CC). Based on the GO results, GO terms with *P* < 0.05 and *Q* < 0.05 were selected and displayed in bubble plots. Similarly, the enriched Kyoto Encyclopedia of Genes and Genomes (KEGG) pathways were screened with *P* < 0.05 and *Q* < 0.05. For further evaluation of the potential pathways, REACTOME pathways were carefully enriched by Gene Set Enrichment Analysis (GSEA) with *P* < 0.05 and FDR < 25%.

### The correlation between the m7G cluster and immune checkpoints, immune infiltration, TCIA, immunotherapy response, and chemotherapy response

We analyzed and compared the expression of 20 checkpoints between the clusters. For estimation of immune cells in TCGA BC samples, the CIBERSORTx website (https://cibersortx.stanford.edu) was used to calculate the contents of 22 types of immune cells in BC samples. Then, we compared the contents of infiltrated immune cells between the clusters.

To further verify the above results, based on the TCGA BC cohort, the Tumor Immune Dysfunction and Exclusion (TIDE) algorithm was used to predict and compare the immunotherapy response between the cluster 1 and cluster 2. Meanwhile, we compared the RNA-seq and clinical data of BC patients receiving immunotherapy in the real world. The patients in the IMvigor210 cohort were subtyped by the R packages “ConsensusClusterPlus” and “limma” using the above four genes. Then, the IMvigor210 trial (EGAD00001003977), including patients with advanced or metastatic BC who were treated with an anti-PD-L1 agent (atezolizumab), was employed to assess the correlation between therapeutic outcomes and the clusters [23]. Moreover, Genomics of Drug Sensitivity in Cancer (GDSC), a public pharmacogenomics database, was used to predict various chemotherapy responses for BC patients. The half-maximal inhibitory concentration (IC50) calculated by the “pRRophetic” package in R software was the main endpoint of the chemotherapy response and drug sensitivity, which was also compared between the clusters.

### Statistical analysis

According to the normality and quality of variances of the data, one-way ANOVA or the Mann‒Whitney *U* test was used to perform statistical analysis of three or more continuous variables. Quantitative data in two groups were compared using Student’s *t* test. All analyzed data are displayed as the standard deviation (SD). A *P* < 0.05 was considered significant for all analyses, which were performed using R version 3.6.3 and relative packages. ^ns^*P* ≥ 0.05; *P < 0.05; **P < 0.01; ***P < 0.001.

## Results

### Gene selection and basal data

**Fig. 1:**
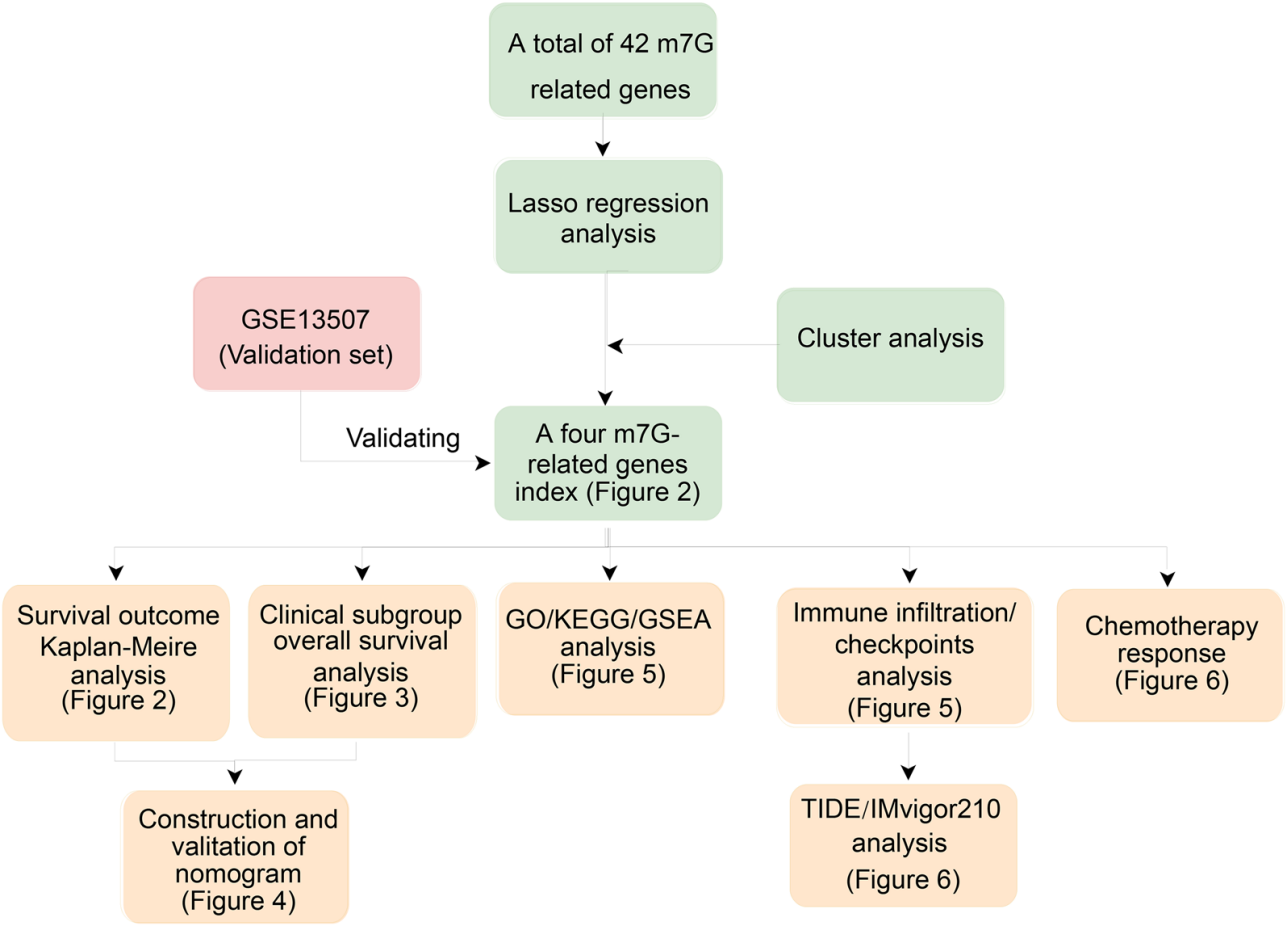
The workflow of this study

**Fig. 2:**
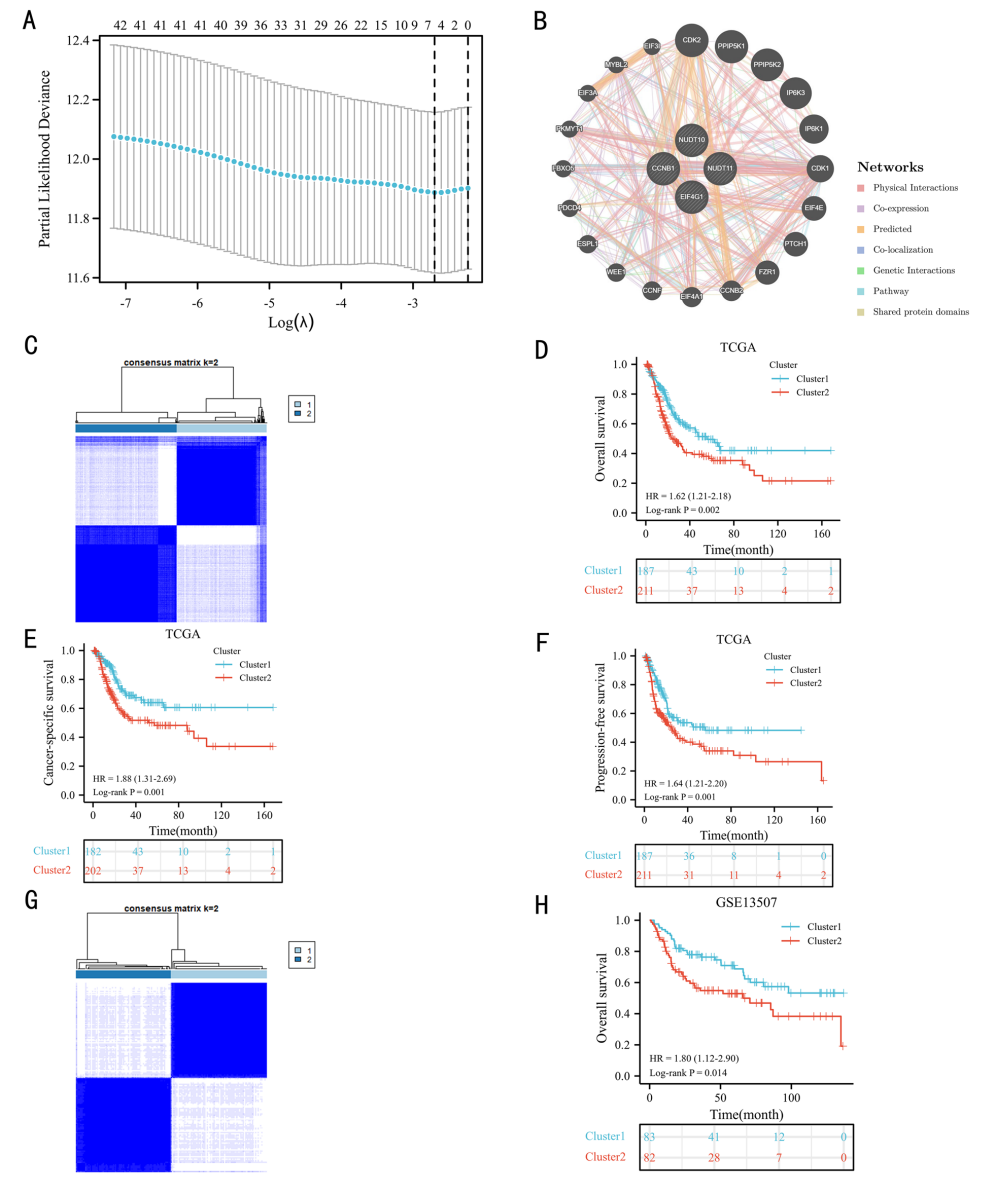
Molecular subtype: the lasso regression model results (**A**), the protein–protein interaction network (**B**); cluster plot showing two distinct groups in the TCGA database (**C**); Validation of the cluster: the Kaplan‒Meier curve results of overall survival (OS) (**D**), cancer-specific survival (**E**) and progression-free survival (**F**) in the TCGA dataset, cluster plot showing two distinct groups in the GSE13507 (**G**), and the Kaplan‒Meier curve results of OS in the GSE13507 dataset (**H**)

The workflow of our study is shown in Fig. 1. The 42 m7G-related genes displayed in Additional file 1: Table S1. As presented in Fig. 2A, the lasso regression model screened EIF4G1, NUDT11, NUDT10, and CCNB1. According to the results of the protein–protein interaction network, the four genes were mainly associated with CDK2, PPIP5K1, PPIP5K2, IP6K3, IP6K1, and CDK1 (Fig. 2B). Finally, these four m7G-related genes divided BC patients in the TCGA dataset into two clusters (Fig. 2C, consensus matrix *k* = 2). The expression of these four genes in the m7G-related clusters is shown in Additional file 2: Fig. S1. In the TCGA dataset, patients in the cluster 2 had worse OS (Fig. 2D, *P* = 0.002), CSS (Fig. 2E, *P* = 0.001), and PFS (Fig. 2F, *P* = 0.001) than those in the cluster 1. Similarly, patients in the GSE13507 dataset were classified into two clusters by the four m7G-related genes (Fig. 2G, consensus matrix *k* = 2). In the GSE13507 cohort, patients in the cluster 2 were significantly correlated with worse OS than patients in the cluster 1 (Fig. 2H, *P* = 0.014).

The basic information of the TCGA and GSE13507 datasets is displayed in Table 1 and Additional file 3: Table S2, respectively. In TCGA dataset, the cluster 2 was significantly associated with WHO high grade, poor OS, CSS, and PFS (Table 1).

**Table 1:** The clinicopathological characteristics of the TCGA included patients

Characteristic	Cluster 1	Cluster 2	*P*
*n*	187	211	
Age, mean ± SD	67.46 ± 11.29	68.11 ± 9.79	0.536
BMI, mean ± SD	27.1 ± 5.78	26.84 ± 5.57	0.670
Sex, *n* (%)			0.271
Female	44 (11.1%)	61 (15.3%)	
Male	143 (35.9%)	150 (37.7%)	
Smoking history, *n* (%)			0.071
No	124 (32.2%)	156 (40.5%)	
Yes	58 (15.1%)	47 (12.2%)	
Lymphatic vessel invasion, *n* (%)			0.913
Yes	56 (20.7%)	67 (24.8%)	
No	69 (25.6%)	78 (28.9%)	
WHO grade, *n* (%)			< 0.001
High	170 (43%)	207 (52.4%)	
Low	16 (4.1%)	2 (0.5%)	
AJCC stage, *n* (%)			0.114
AJCC stage III–IV	67 (16.9%)	59 (14.9%)	
AJCC stage I–II	119 (30.1%)	151 (38.1%)	
Distant metastasis, *n* (%)			0.528
M0	92 (45.5%)	100 (49.5%)	
M1	6 (3%)	4 (2%)	
Lymph node metastasis, *n* (%)			0.902
N+	58 (16.2%)	69 (19.3%)	
N0	108 (30.3%)	122 (34.2%)	
T stage, *n* (%)			0.347
T2_4	112 (30.5%)	137 (37.3%)	
Ta_1	60 (16.3%)	58 (15.8%)	
Overall survival, *n* (%)			0.005
Alive	119 (29.9%)	104 (26.1%)	
Dead	68 (17.1%)	107 (26.9%)	
Cancer-specific survival, *n* (%)			0.003
Alive	139 (36.2%)	125 (32.6%)	
Dead	43 (11.2%)	77 (20.1%)	
Progression-free survival, *n* (%)			0.005
No	120 (30.2%)	105 (26.4%)	
Yes	67 (16.8%)	106 (26.6%)	

*AJCC*, American Joint Committee on cancer; *BMI*, body mass index; *SD*, Standard deviation; *WHO*, World Health Organization; *n*, Number

### Validation of the m7G-related cluster

**Fig. 3:**
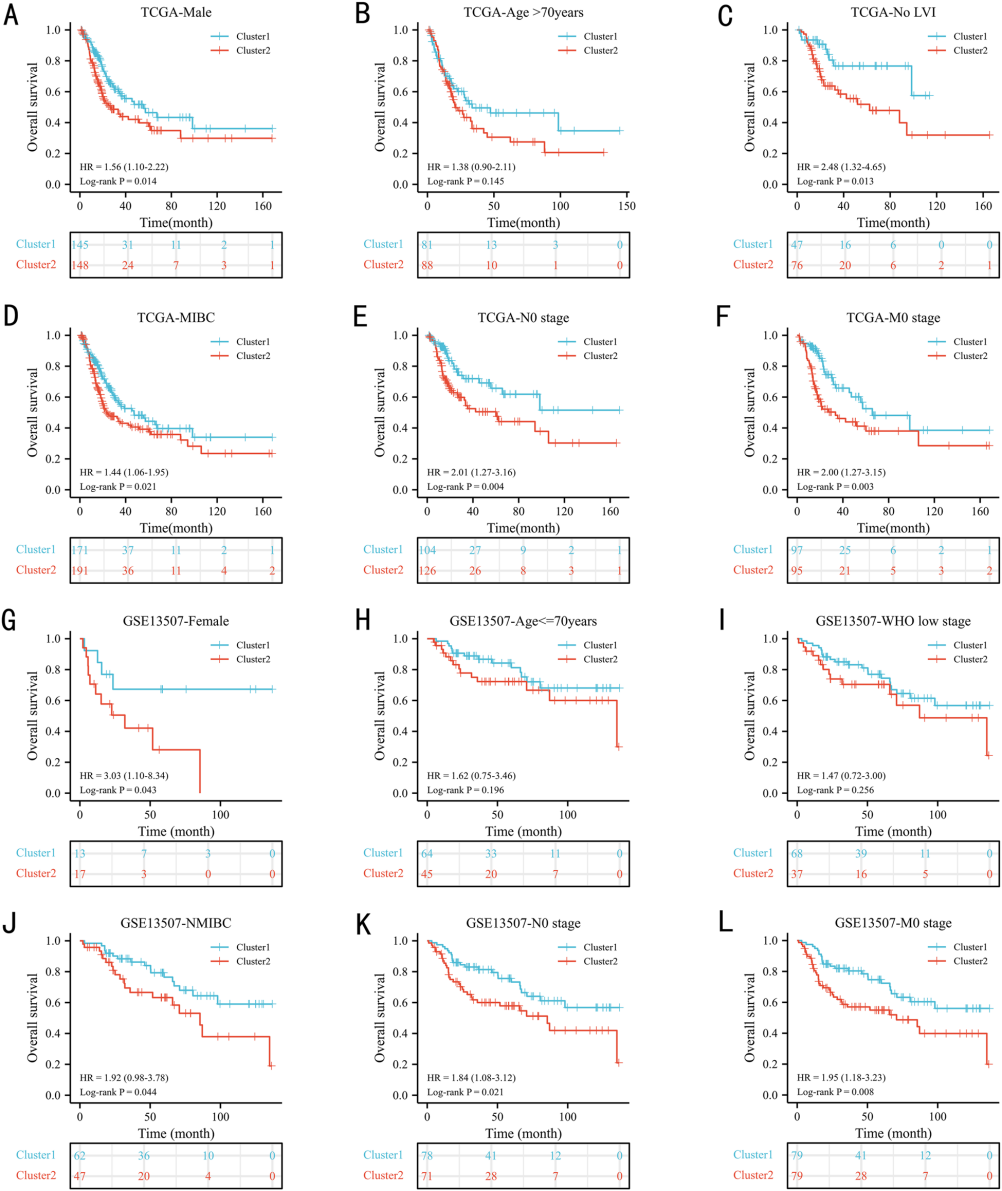
The prognostic ability of the cluster in clinical subgroups: Kaplan‒Meier analysis result of subgroups in TCGA database: male (**A**), age > 70 years (**B**), no lymphatic vessel invasion (**C**), T2_4 stage (**D**), no lymph node metastasis (**E**), no distant metastasis (**F**); in the GSE13507 dataset: female (**G**), age ≤ 70 years (**H**), were low grade (**I**), Ta_1 (**J**), no lymph node metastasis (**K**), no distant metastasis (**L**). N: lymph node metastasis; M: distant metastasis; LVI: lymphatic vessel invasion; WHO: World Health Organization

The Kaplan‒Meier curves was utilized to estimate the prognostic value of the cluster in clinical subgroups. In the TCGA database, the cluster exhibited significant prognostic value in the male (Fig. 3A, *P* = 0.014), no lymphatic vessel invasion (Fig. 3C, *P* = 0.013), T2_4 stage (Fig. 3D, *P* = 0.021), no lymph node metastasis (Fig. 3E, *P* = 0.004), and no distant metastasis (Fig. 3F, *P* = 0.003) subgroups, except for the age > 70 years (Fig. 3B, *P* = 0.145) subgroup. In the GSE13507 dataset, the cluster exhibited significant prognostic value in the female (Fig. 3G, *P*  = 0.043), Ta_1 stage (Fig. 3J, *P* = 0.044), no lymph node metastasis (Fig. 3K, *P* = 0.021), and no distant metastasis (Fig. 3L, *P*  = 0.008) subgroups. No significant differences were found between the cluster 1 and cluster 2 in the age ≤ 70 years (Fig. 3H, *P* = 0.196) and WHO low-grade (Fig. 3I, *P* = 0.256) subgroups.

### Construction and validation of a nomogram

**Fig. 4:**
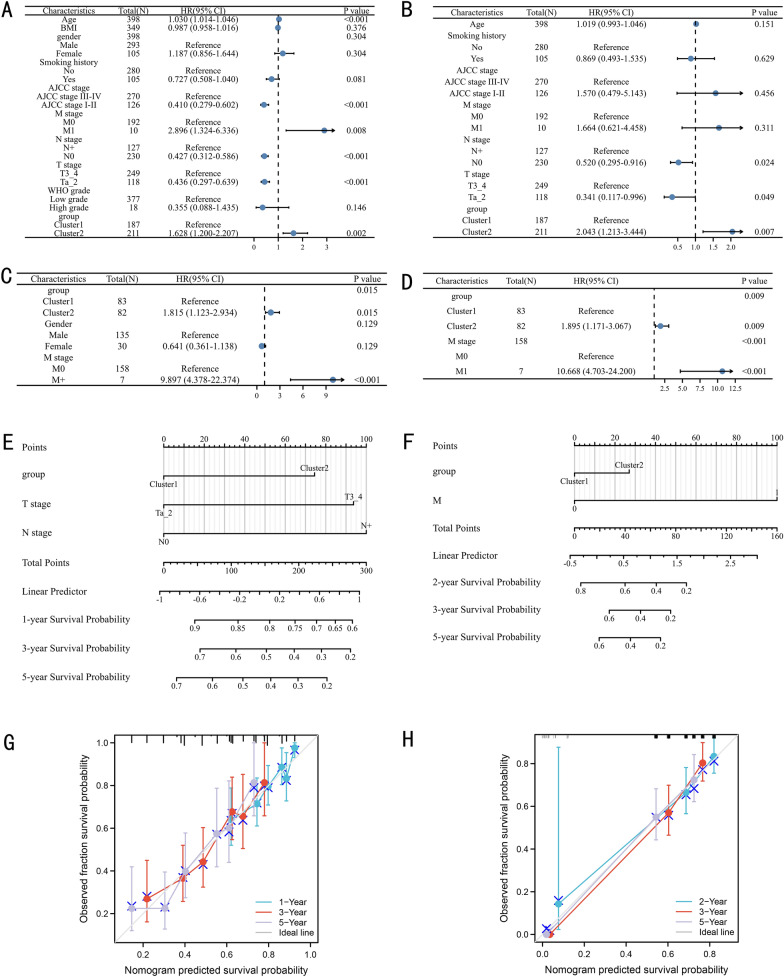
Construction and validation the nomogram: univariable (**A**) and multivariable (**B**) Cox regression model in the TCGA database, univariable (**C**) and multivariable (**D**) Cox regression model in the GSE13507 dataset, the nomograms based on the TCGA (**E**) and GSE13507 datasets (**F**), The calibration curves of the TCGA nomogram (**G**) and GSE13507 nomogram (**H**). N: lymph node metastasis; M: distant metastasis; WHO: World Health Organization

Univariate Cox regression model was used to analyze the prognostic value of the cluster and clinical parameters in the TCGA (Fig. 4A) and GSE13507 (Fig. 4C). Then, the multivariate Cox regression models consisted of factors with *P* < 0.1 in these two datasets. The results identified that the cluster could independently predict the prognosis of BC patients in the TCGA (Fig. 4B, *P* = 0.007) and GSE13507 (Fig. 4D, *P* = 0.009) datasets. According to the results of the multivariate Cox regression models, we constructed two nomograms based on the factors with *P* < 0.05 in the TCGA (Fig. 4E) and GSE13507 (Fig. 4F). The TCGA nomogram had a C-index of 0.654 (0.632–0.677). In the GSE13507 nomogram, the C-index was 0.645 (0.614–0.677). In the TCGA nomogram, the calibration curves showed good agreement between the predicted value and the true value at 1, 3, and 5 years (Fig. 4G). Similarly, the calibration curves of GSE13507 also showed good agreement between the predicted value and the true value at 2, 3, and 5 years (Fig. 4H).

### The results of GO, KEGG, and gene set enrichment analyses

**Fig. 5:**
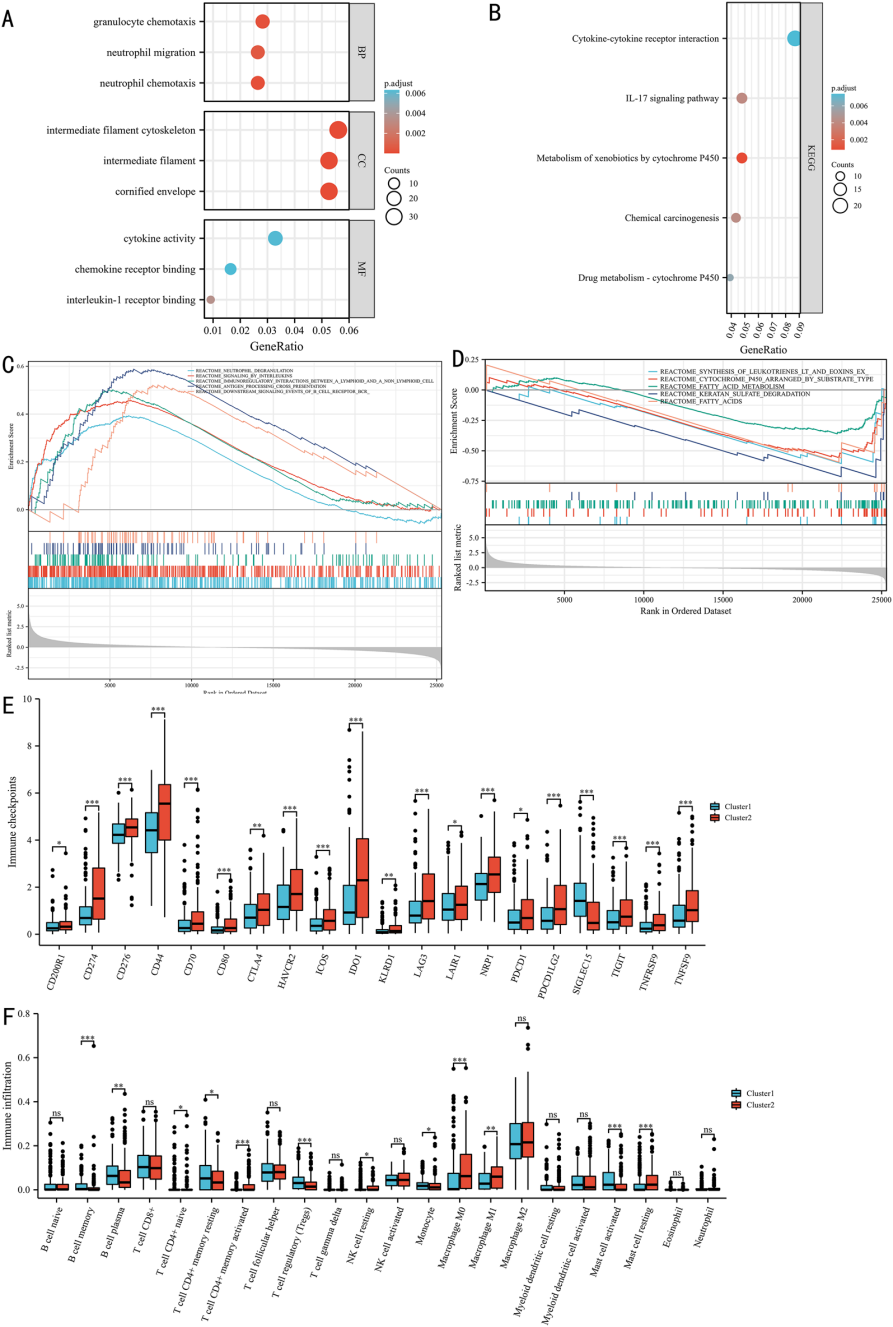
Functional analysis: the Gene Ontology results (**A**), Kyoto Encyclopedia of Genes and Genomes results (**B**), Gene Set Enrichment Analysis results (**C, D**), immune checkpoint analysis results (**E**), immune infiltration (**F**). ^ns^*P* ≥ 0.05; *P < 0.05; **P < 0.01; ***P < 0.001

The data from the TCGA dataset were used to perform GO, KEGG, and GSEA analyses. Figure 5A presents the GO results, showing that the cluster was associated with neutrophil chemotaxis, granulocyte chemotaxis, neutrophil migration in BP, cornified envelope, intermediate filament, intermediate filament cytoskeleton in CC, interleukin-1 receptor binding, cytokine activity, and chemokine receptor binding in MF. According to the KEGG results, the cluster was significantly involved in the IL-17 signaling pathway, chemical carcinogenesis, drug metabolism—cytochrome P450, cytokine‒cytokine receptor interaction, and metabolism of xenobiotics by cytochrome P450 pathways (Fig. 5B). In further GSEA, the cluster was significantly involved in immune-associated pathways, such as neutrophil degranulation, signaling by interleukins, immunoregulatory interactions between lymphoid and nonlymphoid cells, antigen processing cross-presentation, and downstream signaling events of b cell receptor BCR pathways (Fig. 5C). Moreover, the cluster also enriched metabolism-associated pathways, including synthesis of leukotrienes LT and eoxins ex, cytochrome p450 arranged by substrate type, fatty acid metabolism, keratan sulfate degradation, and fatty acids pathways (Fig. 5D).

### The correlation between the risk score and immune checkpoints and immune cell infiltration

Based on TCGA data, we identified the presentation of the cluster in immune-related analysis. As shown in Fig. 5E, the cluster 2 was positively correlated with many immune checkpoints, such as CD274, CTLA4, HAVCR2, LAG3, PDCD1, and PDCD1LG2, while SIGLEC15 was highly expressed in the cluster 1. The results, as shown in Fig. 5F, indicated that samples in the cluster 2 had highly activated CD4+ memory T cells, NK resting cells, M0 macrophages, M1 macrophages, and resting mast cells infiltration. In the cluster 1, samples had more B memory cells, B plasma cells, resting memory CD4+ T cells, T regulatory cells, monocyte cells, and activated mast cells infiltration.

### The correlation between the m7G-related risk score and immunotherapy and chemotherapy

**Fig. 6:**
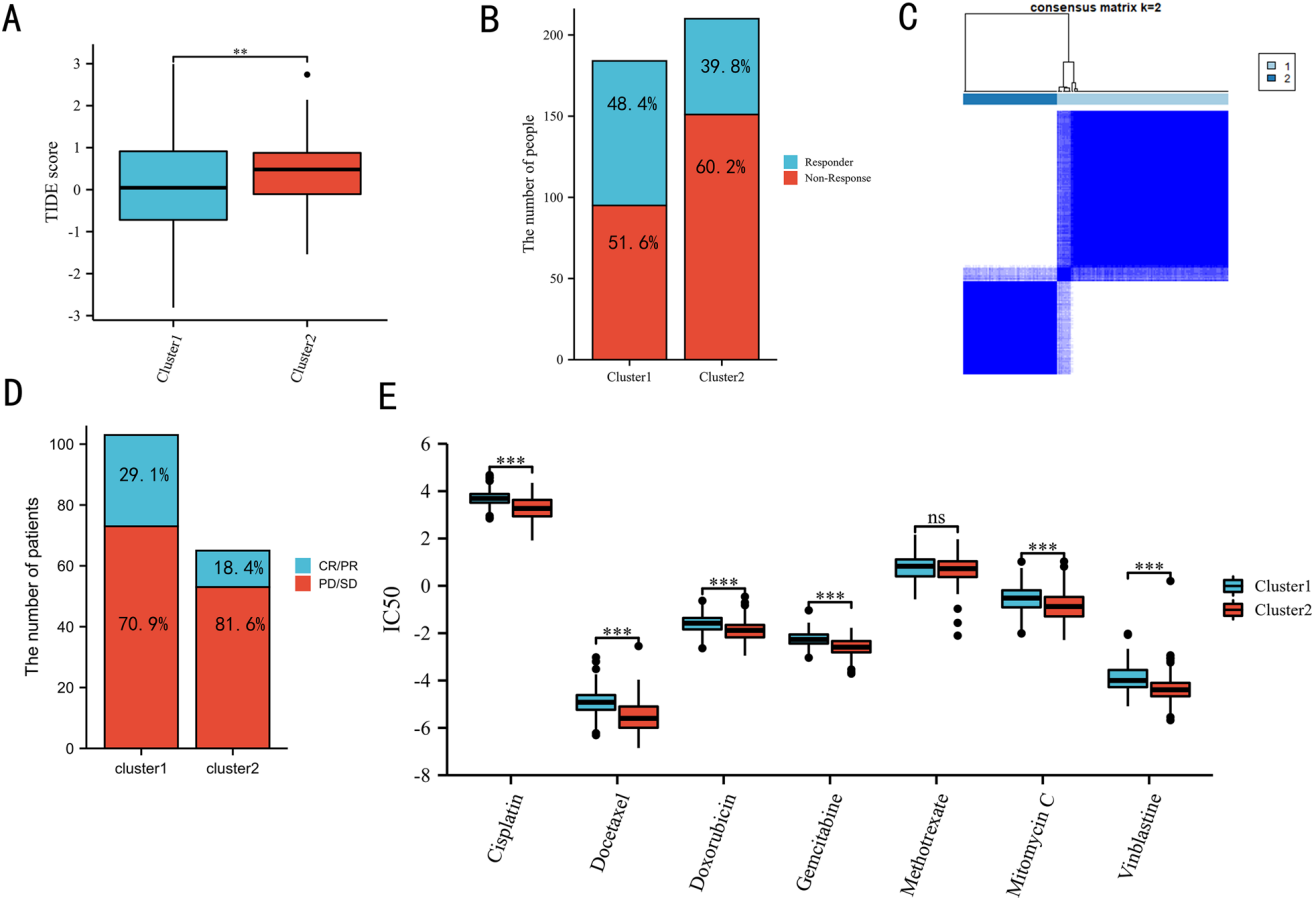
The correlation between the cluster and immunotherapy response: the correlation of the TIDE score and the clusters (**A**), TCGA patients with/without immunotherapy response between the clusters (**B**), cluster plot showing two distinct groups in the IMvigor210 cohort (**C**), IMvigor210 patients with/without immunotherapy response in the clusters (**D**). Chemotherapy response (J). IC50: half-maximal inhibitory concentration, CR: complete response, PR: partial response, SD: stable disease, PD: progressive disease. ^ns^*P* ≥ 0.05; *P < 0.05; **P < 0.01; ***P < 0.001

As shown in Fig. 6A, the cluster 2 had a significantly higher TIDE score than the cluster 1 (*P* < 0.01). Meanwhile, the response rate of cluster 1 was 48.4%, which was higher than that of cluster 2 (39.8%) (Fig. 6B). In accordance with the TCGA and GSE13507 datasets, patients in the IMvigor210 cohort were also classified into two clusters (Fig. 6C, consensus matrix *k* = 2). According to the therapeutic outcome of IMvigor210, the response rate of cluster 1 was 29.1% (30/73), which was higher than the 18.4% (12/53) of cluster 2 (Fig. 6D). Both the TIDE and IMvigor210 results supported that patients in the cluster 1 were more likely to benefit from immunotherapy. In chemotherapy, patients in cluster2 were significantly sensitive to cisplatin, docetaxel, doxorubicin, gemcitabine, mitomycin C, and vinblastine based on the TCGA dataset (Fig. 6E, all *P* < 0.001).

## Discussion

As the key role of RNA modification, m7G is involved in the processing, metabolism, and function of mRNAs, microRNAs (miRNAs), tRNAs, and rRNAs [13]. Recently, increasing evidence has found that m7G-related genes can promote or inhibit the processes of various cancers [14, 19, 21, 24–26]. Therefore, this study orchestrated molecular subtypes and identified key genes for BC from the perspective of m7G. In our results, the m7G-related cluster could independently predict the prognosis of BC patients. Furthermore, there was a significant correlation between the cluster and immunotherapy and chemotherapy. These results suggested that the m7G-related cluster mainly paved a roadmap for precision medicine.

As a part of a scaffold component of eukaryotic translation initiation factor 4F, eukaryotic translation initiation factor 4G 1 (EIF4G1) is closely associated with various tumors, especially in solid tumors [27]. Nudix hydrolase 10 (NUDT10), located in Xp11.22, increases promoter methylation and is associated with OS in prostate cancer and gastric cancer [28, 29]. NUDT10 and Nudix hydrolase 11 (NUDT11) can promote prostate carcinogenesis and increase prostate cancer susceptibility by being involved in a variety of biological processes and mediating cellular stress responses [30]. CyclinB1 (CCNB1) is mainly involved in cell cycle regulation and is associated with the epithelial–mesenchymal transition (EMT) process [31]. In BC, CCNB1 is an oncogene and could predict the prognosis of nonmuscle-invasive patients [31].

In this study, we validated the prognostic value of the cluster in OS, CSS, and PFS for BC patients in the TCGA dataset. For further estimation, the GSE13507 cohort was used to identify the stability of this result, which also suggested that the cluster could independently predict the prognosis of BC patients. This finding was also reported by Ye et al. [19]. They also identified that the m7G-related signature could predict the prognosis of prostate cancer. Similarly, Liu et al. [20] also identified the prognostic value of m7G-related lncRNAs in prostate cancer. Moreover, three studies demonstrated the prognostic value of m7G-related mRNAs, lncRNAs, and miRNAs in clear cell renal cell carcinoma [16–18]. Furthermore, we also assessed the prognostic value of the risk score in various subgroups. The subgroup results identified that the risk score could predict the prognosis of BC patients in TCGA and external validation cohorts. Thus, we speculate that the m7G-related cluster might be a potential biomarker for BC.

After assessing the prognostic value, we tried to explore the function of the m7G-related cluster. The GO, KEGG, and GSEA enrichment results indicated that the cluster was significantly associated with immune- and metabolism-associated pathways. In detail, the pathways involved in the regulation of immune factors, such as neutrophil degranulation and interleukins. Furthermore, these pathways could also influence the regulation of immune cells. Recently, a study reported that the cap structure of m7G can induce immune evasion by enhancing the identity of RNA as a ‘self-molecule’ for cellular recognition factors [32]. This finding might explain why the cluster was mainly involved in immune-related pathways. These results inspired us to verify the correlation between the cluster and immunotherapy. In immune checkpoint analysis, CD274, CTLA4, HAVCR2, LAG3, PDCD1, PDCD1LG2, etc., while SIGLEC15 was highly expressed in cluster 1. No consensus has been reached on the predictive value of immune checkpoints in BC immunotherapy, such as PD-L1 and CTLA4 [33]. In theory, an immunosuppressive microenvironment promotes the expression of immune checkpoints, as presented in the present study. To validate this hypothesis, we performed immune infiltration analysis. The results revealed that samples in cluster 2 had high NK resting cell, macrophage M0 cell, macrophage M1 cell, and mast resting cell infiltration. Of these, macrophages consist of 3 classes: M0, M1, and M2 macrophages. M0 macrophages can differentiate into M1 or M2 macrophages and were significantly associated with poor survival, which was consistent with our study [34]. Based on the results of basic experiments, Bopp et al. [35] has reported that tumor-associated macrophages can promote immune evasion and tumor growth by expressing G-protein-coupled receptors. In addition to assessing the institution of the immune checkpoint and the tumor microenvironment, we also adopted the TIDE score to predict the response rate between cluster 1 and cluster 2. The immunotherapy response rate was negatively associated with the TIDE score [36]. In the TIDE results, culster1 was significantly correlated with a lower TIDE score than cluster 2, which was consistent with the previous hypothesis that patients in cluster 1 were more likely to benefit from immunotherapy. For further validation of the results with real-world data, IMvigor210, including patients with advanced or metastatic BC who were treated with an anti-PD-L1 agent (atezolizumab), was employed to compare the therapeutic outcomes between cluster 1 and cluster 2. In accordance with the above results, a higher response rate was presented in cluster 1 patients. Taken together, we believe that patients in cluster 1 are more likely to benefit from immunotherapy, at least from anti-PD-L1 therapy. In addition to immunotherapy, we also explored the predictive function of the cluster in chemotherapy. In chemotherapy, patients in cluster 2 were associated with low IC50 values of cisplatin, docetaxel, doxorubicin, gemcitabine, mitomycin C, and vinblastine, which suggested that patients in cluster 2 might accept chemotherapy rather than immunotherapy. This evidence suggested that we successfully determined molecular subtypes and identified key genes for BC from the perspective of m7G to pave a roadmap for the evolution of immunotherapy and precision medicine.

A few limitations should be noted. Although we identified the expression and prognostic value of the signature in TCGA and external cohorts, basic experiments should also be performed, which is the next work of us. In this study, we also found some sensitive drugs to the four investigated genes, which need to be further studied in vivo and in vitro.

## Conclusion

The results in TCGA and external validation cohorts identified that we successfully determined molecular subtypes and identified key genes for BC from the perspective of m7G, thereby paving a roadmap for the evolution of immunotherapy and precision medicine.

## Supplementary Information

**Additional file 1: Table S1.** M7G-related genes.

**Additional file 2: Figure S1.** Comparison of four genes expression between the cluster1 and cluster2 in the TCGA dataset (A) and GSE13507 dataset (B).

**Additional file 3: Table S2.** The clinicopathologic characteristics of the GSE13507 included patients.

## Data Availability

All data from this study were downloaded from online databases. Therefore, everyone can get the data online. Further inquiries can be directed to the corresponding author.

## Abbreviations

WDR4: WD Repeat Domain 4
NUDT10: Nudix Hydrolase 10
CCNB1: Cyclin B1
BC: Bladder cancer
TCGA: The Cancer Genome Atlas
GSEA: Gene Set Enrichment Analysis
GO: Gene Ontology
m7G: N7-methylguanosine
RC: Radical cystectomy
METTL1: Methyltransferase-like 1
OS: Overall survival
CSS: Cancer-specific survival
PFS: Progression-free survival
KEGG: Kyoto Encyclopedia of Genes and Genomes
GDSC: Genomics of Drug Sensitivity in Cancer
