# Supplementary figures and images for: M7G-related molecular subtypes can predict the prognosis and correlate with immunotherapy and chemotherapy responses in bladder cancer patients
Source: Eur J Med Res. 2023 Feb 2;28:55. doi: 10.1186/s40001-023-01012-x (PMC9893617; doi:10.1186/s40001-023-01012-x)

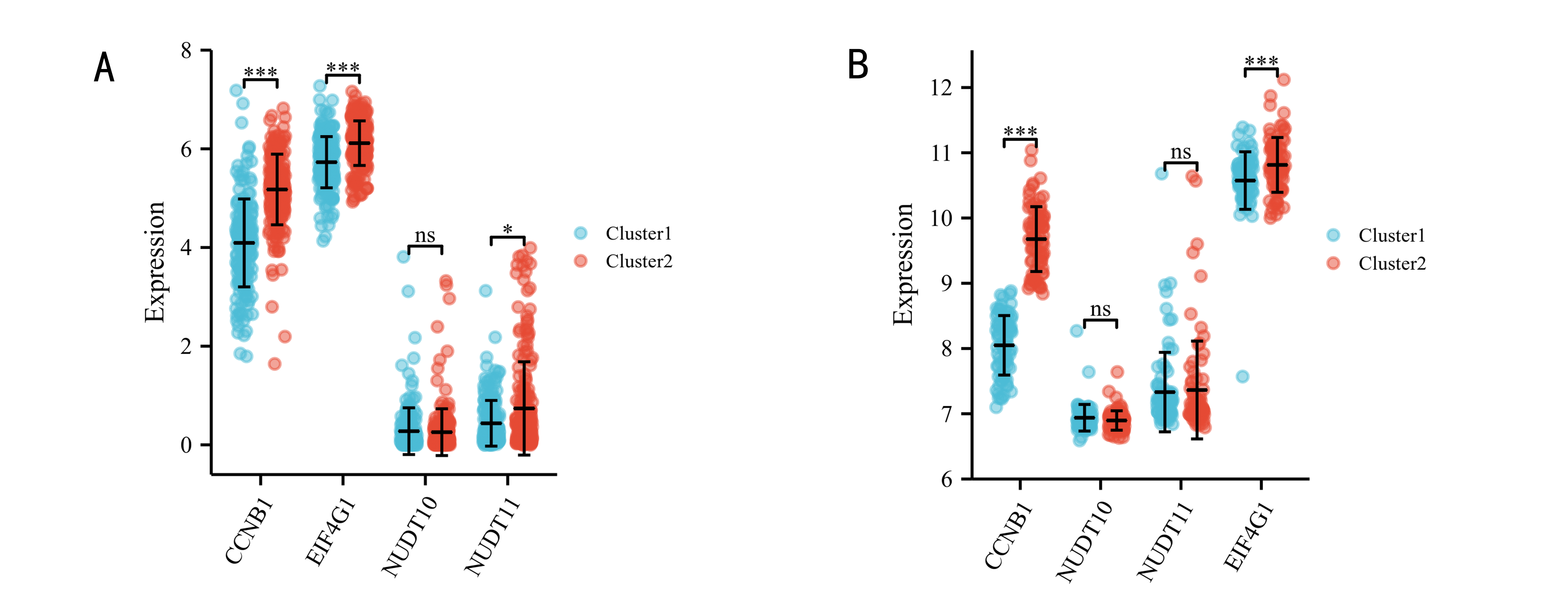

Supplement: Supplementary file 2 — Additional file 2: Figure S1. Comparison of four genes expression between the cluster1 and cluster2 in the TCGA dataset (A) and GSE13507 dataset (B). [file 40001_2023_1012_MOESM2_ESM.tif]
