# Supplementary material for: M7G-related molecular subtypes can predict the prognosis and correlate with immunotherapy and chemotherapy responses in bladder cancer patients
Source: Eur J Med Res. 2023 Feb 2;28:55. doi: 10.1186/s40001-023-01012-x (PMC9893617; doi:10.1186/s40001-023-01012-x)
Supplement: Supplementary file 3 — Additional file 3: Table S2. The clinicopathologic characteristics of the GSE13507 included patients. [file 40001_2023_1012_MOESM3_ESM.docx]

Supplementary Table 2. The clinicopathologic characteristics of the GSE13507 included patients.

| Characteristic | Cluster1 | Cluster2 | p |
| --- | --- | --- | --- |
| n | 83 | 82 |  |
| Age, mean ± SD | 62.4 ± 12.12 | 68 ± 11.2 | 0.002 |
| Sex, n (%) |  |  | 0.673 |
| Female | 13 (7.9%) | 17 (10.3%) |  |
| Male | 70 (42.4%) | 65 (39.4%) |  |
| T stage, n (%) |  |  | 0.003 |
| T2_4 | 21 (12.7%) | 40 (24.2%) |  |
| Ta_1 | 62 (37.6%) | 42 (25.5%) |  |
| Lymph node metastasis, n (%) |  |  | 0.077 |
| N0 | 78 (47.3%) | 71 (43%) |  |
| N+ | 5(3%) | 11 (6.6%) |  |
| WHO grade, n (%) |  |  | < 0.001 |
| Low | 68 (41.2%) | 37 (22.4%) |  |
| High | 15(9.1%) | 45 (27.3%) |  |
| Distant metastasis, n (%) |  |  | 1.000 |
| M0 | 79 (47.9%) | 79 (47.9%) |  |
| M1 | 4 (2.4%) | 3 (1.8%) |  |
| Overall survival, n (%) |  |  | 0.073 |
| Alive | 54 (32.7%) | 42 (25.5%) |  |
| Dead | 29 (17.6%) | 40 (24.2%) |  |

AJCC: American Joint Committe on cancer; BMI: body mass index; SD: Standard deviation; WHO: World Health Organization; n: Number.
